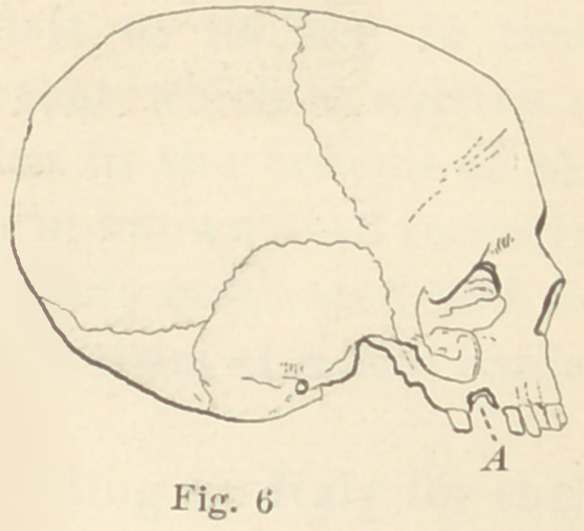# Some Evidences of Prehistoric Dentistry in Italy

**Published:** 1885-01

**Authors:** J. G. Van Marter

**Affiliations:** Rome, Italy


Specimen of Ancient Etrurian and Roman Dentistry.

THE

### Independent Practitioner.

1183.

VOL. VI. JANUARY, 1885. NO. 1.

### Original Communications.

### SOME EVIDENCES OF PREHISTORIC DENTISTRY IN ITALY.

BY J. G. VAN MARTER, A. B.; D. D. S., ROME, ITALY.

When, several years ago, I began to study the vestiges of the race of men which preceded the great Roman people, little did I think that I should find evidences of an advanced stage of dentistry among the relics of that wonderful prehistoric nation. Up to that time I had never found anything in dental literature to assist or guide me into the Etrurian *terra incognita*, but I determined to explore this archæological dark continent in search of evidence of dental weakness. I was aware that the task would not be an easy one, for the Etruscans, once a great and powerful nation, far advanced in civilization, science, and the arts, have been an extinct race for more than two thousand years. They left us no key to their strange language, and no history except that which is written in their tombs. But from these tombs, hidden in the bosoms of the earth for so many centuries, and practically unknown until recently, we now obtain conclusive evidence of a highly cultivated and intelligent race of men, and of the influences on them of a still earlier race.

Great credit is due the archæologists residing in Italy for their efforts in opening up these buried cities of the past, and bringing to light all we now know of Etruscan customs, manners, and history.

One of the localites fruitful in discoveries of Etruscan relics of especial interest to me is the city of Corneto-Tarquinius, near Civita

Vecchia, on the Mediterranean coast. Through the kindness of Prof. Dr. Helbig, a German archæologist in Rome, I was enabled, last year, to become acquainted with the mayor of Corneto, Sig. Cav. Dasti, an eminent Italian archæologist. By his kind permission I was afforded every facility for visiting many of the subterranean Etruscan and Roman tombs, with the privilege of examining at my leisure the exceedingly interesting and valuable collection of Etruscan relics in the Corneto Museum. It was in this museum that I found, carefully guarded with lock and key, the two specimens of ancient dentistry, the drawings of which are here presented. (See Plate 2). That there may be no gainsaying the authenticity of these specimens of ancient Etruscan and Roman dentistry, I procured from Sig. Cav. Dasti, who is the royal inspector of excavations at Corneto, a certificate, duly signed and sealed, of the date of the opening of the tombs in which these dental relics were found. I do not include it in this article, for want of space. I am assured by Prof. Dr. Helbig, who is an undoubted authority, that the tombs from which these early relics of dentistry were taken were virgin tombs, which date back four and five hundred years before the Christian era.

To enable the reader to understand how carefully the Etruscans guarded their dead, I have had drawings made of a family tomb as it appeared two thousand five hundred years ago, before time had smoothed these wrinkles off the face of the earth. (See Plate 1.) The Etruscan tomb here delineated is a probably correct restoration of one of the ancient sepulchres at Corneto-Tarquinius. The necropolis in which this was situated occupied a large extent of territory, which was covered with similar tombs, some larger and some smaller. The average length and size may be inferred from the relative size of the entrance door, which was sufficient to easily admit a full-grown man. In those which I have visited one descends from twenty to forty steps below the surface of the earth, into the vault beneath, which is a room varying in size in different tombs, from ten feet square and eight feet high, to thirty feet square and ten feet in height. Many of these vaults were beautifully finished inside, and the walls were ornamented with fine frescoes, representing triumphal marches, games, dancing girls, and domestic scenes. Portions of these frescoes remain bright and beautiful to this day. Naturally, time has leveled the tombs to dust, and for ages the ground has been cultivated above, while the vaults beneath were forgotten and undisturbed until a few years since. The present Italian government has made extensive excavations, and formed a very interesting museum from the relics there found.

It was beneath the ruins of one of these Etruscan tombs that the partial denture, which I shall designate as No. 1, was found. As will be seen by the drawing, No. 1 represents the front view of an arrangement for holding in position three superior artificial teeth, by banding them to adjoining natural teeth. In this drawing the cuspid and lateral incisors were natural teeth, while the two central incisors were evidently carved from some large animal's tooth, to fit the space. Figure No. 2 represents No. 1 in a position to show the missing artificial first bicuspid, and the adjoining natural teeth, which had crumbled to dust when this relic of human misery was unearthed. No. 3 represents a partial denture which was taken from an ancient Roman tomb, dating back four hundred years B. C. The remaining tooth in this specimen was evidently a human tooth, as, no doubt, was the missing one. It represents the early Roman method of replacing two inferior incisor teeth on the Etruscan plan. Figure No. 4 shows No. 3 reversed, giving a clear view of the position of the missing artificial tooth, with the manner of holding the same in position. The gold used in these specimens was very soft, evidently made so for the purpose of more easily slipping the rings over the natural teeth, in adjusting the piece in the mouth. The two centrals in No. 1 were well carved, and the dentures were cleverly made.

These are the earliest known essays at dental bridge-work. What conclusions are we to draw from these evidences of early dentistry? In view of the recent discovery of wonderful surgical instruments found in the ruins of Pompeii, instruments that have been re-invented in recent years to meet the demands of modern surgery, one is almost inclined to call a halt before expressing any opinion, and wait a little longer for the excavators to dig up Etruscan or Umbrian telephones, and evidences of railways and steamboats.

In Figure No. 5 is presented in outline an Etruscan skull in my possession, which was taken from one of the ancient tombs at Corneto. It is minus the inferior maxillary, but it shows clearly a missing right superior first molar, and evidence of alveolar abscess at *a*, proving conclusively that Etruscan mortals suffered the pangs of toothache like mortals of to-day. That is one of the luxuries we have inherited with the exquisite specimens of jewelry they left us. In shape and size this skull very closely resembles the early Egyptian heads. That great Egyptologist, Ebers, has proven that they had teachers of dentistry three thousand years ago. Then they must have had a necessity for dentists. When we are able to decipher the Etruscan inscriptions, possibly we may find that these carpet-bag Etruscans went over to Egypt, and obtained Egyptian diplomas without learning the Egyptian language, and then returned and put on foreign airs. I am aware that some will possibly remind me that one or two supposed antique specimens of Etruscan or early Roman dentistry do not prove that there were dentists in Etruria, or that the early Romans had made the same professional advancement that we have. A visit to the museum at Corneto will partially dispel this illusion, and lead one to the belief that there are more things in heaven and earth than are dreamt of in our philosophy. A few months before the late lamented Dr J. Marion Sims died, he was at my home in Rome, and I related to him what I had seen at Corneto. He expressed the greatest surprise and interest in the subject, and afterwards wrote me from Paris, urging me to pursue my researches and prepare them for publication. Last winter the great English surgeon, Sir Spencer Wells, spent an hour at my house, and he also expressed great interest in the matter, and related to me the story of a dentist who practiced in Egypt, and who had seen mummy teeth that had been filled with a kind of fusible metal. The noted English archæologist and writer in Rome, Mr. Forbes, assures me that he has seen mummy teeth that had been filled with gold. A noble Roman princess has told me that she has seen Etruscan teeth that were artificially filled, and has promised to soon open the way for me to see them. Personally, I have not yet seen any evidence of such dental skill, but I do not despair of demonstrating it in the near future. From my limited observations I am not ready to declare that any of the Etruscans died of toothache, or that they had as bad teeth as we see nowadays. On the contrary, I am inclined to think they had better teeth than we have, and that in many respects they were wiser than we are. As a rule, they cremated their dead, and that custom of theirs renders our task a very difficult and tedious one in procuring evidence.

Judging from what I have seen and read, and from what I have learned from scientific men, only great warriors and civilians of distinction were embalmed and laid at rest in the family tomb, in that part of Etruria included in my observations. Naturally, cremation would reduce to ashes the teeth of those entombed in urns, and two or three thousand years of time has accomplished the same end for nearly all those who were embalmed and laid to rest in state. This narrows our limits of research to a small point, and makes it rather surprising that any artificial dental work should come down to us from those remote days.

> "Wondrous and awful are thy silent halls,
> O kingdom of the past;
> There lie the bygone ages in their palls,
> Guarded by shadows vast.
> There all is hushed and breathless,
> Save when some mirage of old error falls
> Earth worshiped once as deathless."
>
> "Thy mighty clamors, wars, and world-noised deeds
> Are silent now in dust;
> Gone like a tremble of the huddling reeds,
> Beneath some sudden gust.
> Thy forms and creeds have vanished,
> Tossed out to wither like unsightly weeds
> From the world's garden banished."

## Figures and Tables

**Figure f1:**
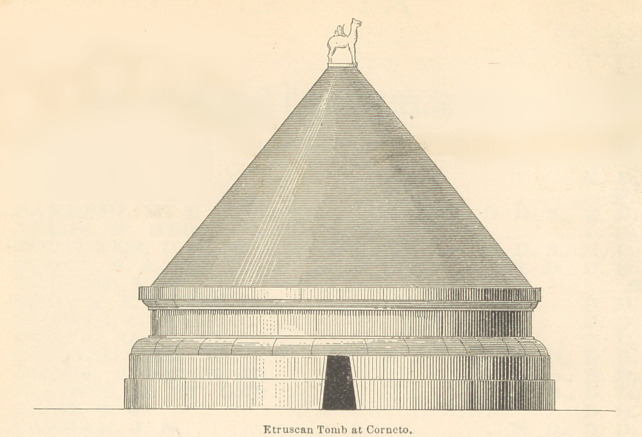


**Figure f2:**
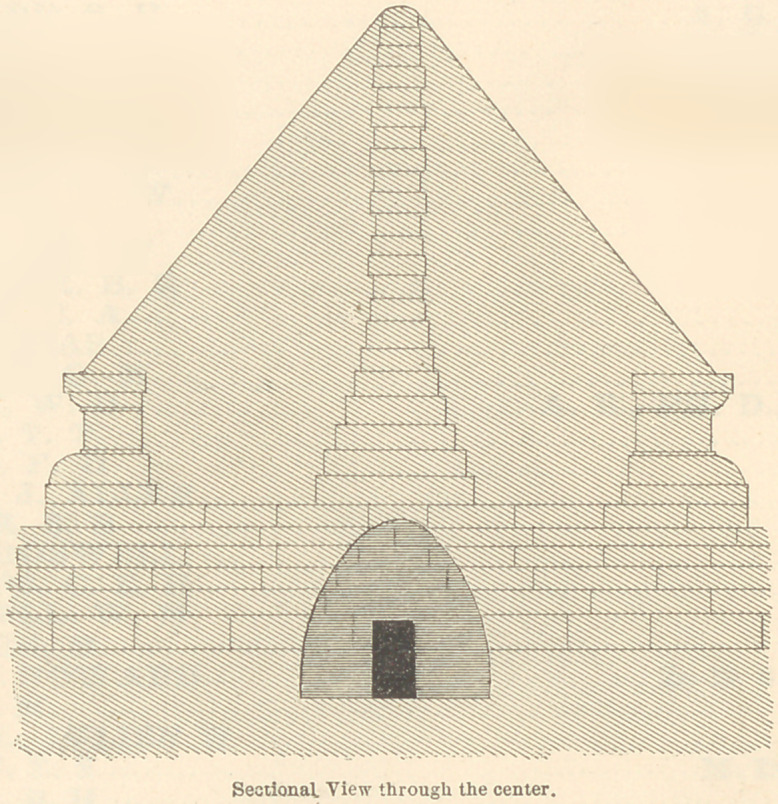


**Figure f3:**
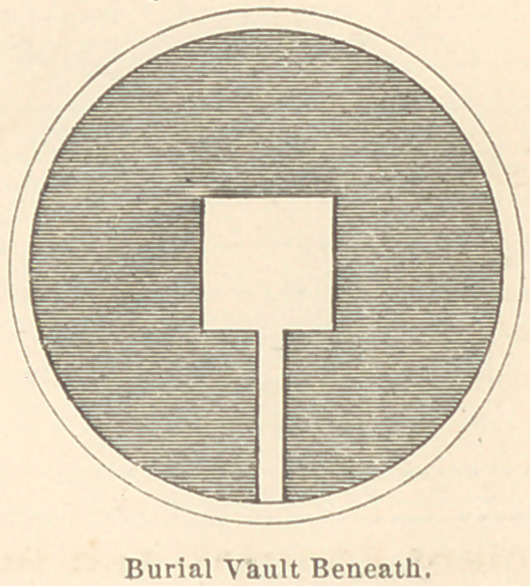


**Fig. 1 f4:**
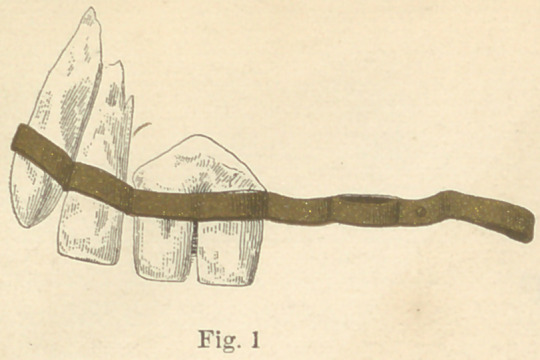


**Fig. 2 f5:**
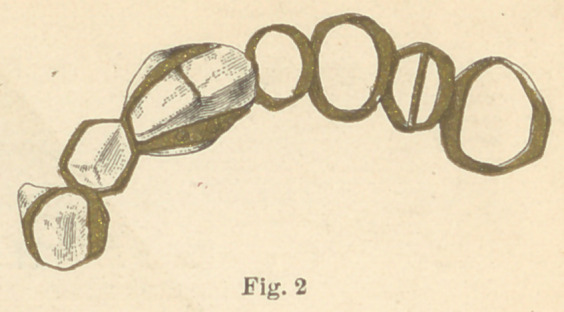


**Fig. 3 f6:**
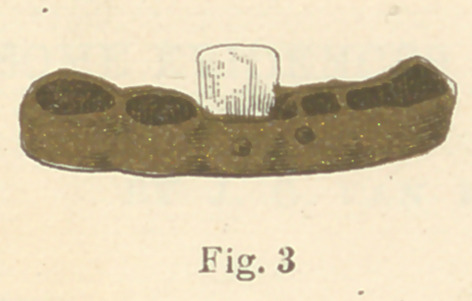


**Fig. 4 f7:**
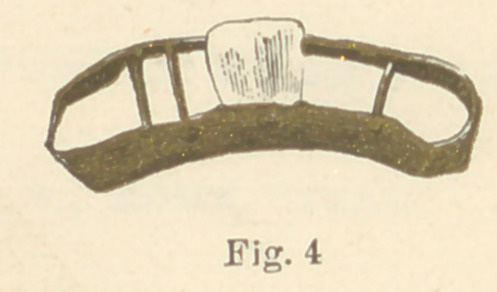


**Fig. 5 f8:**
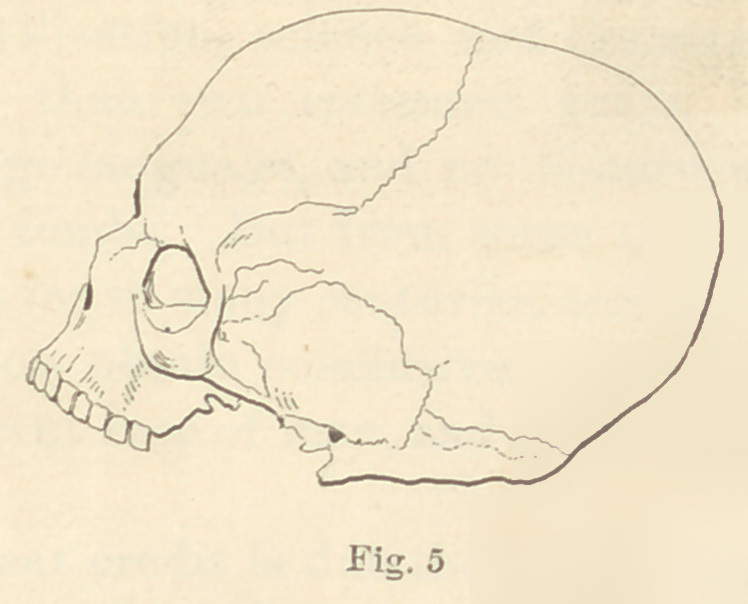


**Fig. 6 f9:**